# An Account of the Late Epidemic Catarrhal Fever, Commonly Called the Influenza, as It Appeared at Bath, in the Months of May and June 1782

**Published:** 1782

**Authors:** 


VIII. *An Account of the late epidemic catarrhal Fever, commonly called the Influenza, as it appeared at Bath, in the months of May and June* 1782. *By* W. Falconer, M. D. F. R. S. 8vo. Dilly, *London.* 28 pages. 1s.

THE ſymptoms of the late epidemic are here deſcribed with great accuracy. It appeared at Bath about the middle of May. It generally began with a ſlight ſhivering, ſucceeded by a ſenſe of ſtuffing in the head and noſe, a diſcharge of a clear, acrid, ſaline fluid from the noſe, and often from the eyes, attended with frequent ſneezing. As the diſorder proceeded, the head became pained and frequently vertiginous. The ſeat of the pain was generally at the fore part of the head.

No external ſoreneſs or ſwelling of the glands took place; nor any internal ulceration of the fauces, in any caſes that fell under our author's obſervations. The pulſe was in general very quick, even to 140 or 150 in a minute, and when the fever ran ſo high, ſome degree of delirium generally prevailed during the night, but conſtantly went off towards morning. A cough generally took place from the beginning, but was ſeldom accompanied with pain in the ſide, or or confiderable interruption of breathing. Thefe fymptoms were commonly attended with confiderable debility. In fome, we are told, a red eruption came on, perhaps caufed by the fweating, towards the decline of the complaint, efpecially about the joints and palms of the hands, but this foon went off without any bad fymptoms.

All ages, our author obferves, were affected with it, from children in the cradle to extreme old age, and the male and female fexes equally. It feldom proved fatal, except to very old perfons, who died as it were fuffocated with the catarrh; or unlefs fome very improper methods had been purfued. They who were recovering from it were extremely liable to relapfes; in which cafe all the fymptoms recurred much in the fame manner as at firft, and fometimes with additional violence.

Dr. Falconer thinks this epidemic bore a great refemblance to the *fcarlatina anginofa*. The feat of both, he obferves, appeared to be the fame, and the fymptoms likewife were very fimilar. The rednefs of the fkin, which is fo general an attendant on the fcarlatina anginofa, was not indeed an ufual fymptom of the late influenza; but it has neverthelefs fometimes appeared, peared, and fomething of this kind, he adds, is mentioned as an attendant on the complaint by other writers.

He fuppofes it to have been of a contagious and inflammatory nature. Mild diaphoretics were found to be the moft efficacious remedies. If the fymptoms were urgent, one bleeding feemed to give fome relief; but, repeated often, it apparently did harm. Emetics were, in fome inftances, of great ufe when given near the acceffion of the diforder. Purging, when carried beyond the mere relief of coftivenefs, feemed to do harm. Blifters had a good effect only where the pain of the head was confiderable; for they never abated either the catarrh or cough, and in feveral inftances feemed to be hurtful by increafing the irritability of the fyftem. The inhalation of warm fteams feemed to aggravate the fymptoms. Opiates were of ufe to leffen the cough and promote expectoration.

No preventatives were of any efficacy in preventing this diforder. The acetum prophylacticum, camphorated fpirits, aromatic waters, and tobacco, were all tried, we are told, with this intent, but without any effect whatever.

